# Evaluation of Sensitivity and Specificity of Three Commercial Real-Time Quantitative Polymerase Chain Reaction Kits for Detecting SARS-CoV-2 in Bangladesh

**DOI:** 10.7759/cureus.20627

**Published:** 2021-12-22

**Authors:** Farzana Mim, Md. Selim Reza, Mohammad Jahidur Rahman Khan, Nurul Karim, Mohammad A Rahman, Md. Ibrahim Hossain, Rajib Biswas

**Affiliations:** 1 Department of Biochemistry and Molecular Biology, Jahangirnagar University, Dhaka, BGD; 2 RT-PCR Lab, Bangabandhu Sheikh Mujib Medical College, Faridpur, BGD; 3 Department of Microbiology, Shaheed Suhrawardy Medical College, Dhaka, BGD; 4 Infectious Diseases Unit, International Centre for Diarrheal Disease Research, Dhaka, BGD

**Keywords:** diagnostic performance, specificity, sensitivity, rt-qpcr, sars-cov-2, covid-19

## Abstract

Background

The coronavirus disease 2019 (COVID-19) pandemic has manifested into an unprecedented public health crisis. The rapid spread of the severe acute respiratory syndrome coronavirus 2 (SARS-CoV-2) has facilitated reagent developers to customize and receive authorization for nucleic acid testing kits in a short period, which would have resulted in some shortcomings in the quality parameters of the kits. Consequently, in-house clinical validations of innovative real-time quantitative polymerase chain reaction (RT-qPCR) kits are required. This research aims to determine the sensitivity, specificity, and accuracy of various RT-qPCR kits available in Bangladesh.

Methodology

A total of 150 samples were obtained from patients with suspected COVID-19 infection when the delta variant was predominant, followed by RNA extraction performed using a nucleic acid isolation kit. Subsequently, three commercially available PCR kits named Sansure (China), STAT-NAT^Ⓑ^ (Sentinel Diagnostics, Italy), and Roche Biochem (Switzerland) were applied to detect SARS-CoV-2.

Results

The results showed that the STAT-NAT^Ⓑ^ kit is more sensitive than the other two, as indicated by the cycle threshold (Ct) values of respective genes. STAT-NAT^Ⓑ^ RT-qPCR can detect the *ORF1ab* gene sensitively (p < 0.001) compared to Sansure. STAT-NAT^Ⓑ^ was also capable of detecting *E* and *RdRp* genes more sensitively (p < 0.001) compared to Roche. Regarding specificity, STAT-NAT^Ⓑ ^(95% confidence interval [Cl] = 92.29-99.73%). RT-qPCR showed more accuracy than Sansure (95% Cl = 90.77-99.32%) and Roche (95% Cl = 81.17-94.38%). The area under the curve for *E*, *ORF1ab*, and *RdRp* genes of the STAT NAT^Ⓑ^ PCR kit was 0.952, 0.959, and 0.981, respectively.

Conclusions

This study concluded that STAT-NAT^Ⓑ^ is a better diagnostic RT-qPCR kit compared to Sansure and Roche for detecting SARS-CoV-2.

## Introduction

The coronavirus disease 2019 (COVID-19) is an infectious disease initially discovered in December 2019 in Wuhan, China [1]. On March 11, 2020, the World Health Organization (WHO) declared COVID-19 a pandemic due to the outbreak of a novel coronavirus named severe acute respiratory syndrome coronavirus 2 (SARS-CoV-2) [2]. SARS-CoV-2 is an enveloped, positive-sense, single-stranded RNA (+ssRNA) virus belonging to the Orthocoronavirinae subfamily of the Coronaviridae family [3]. The first case of SARS-CoV-2 infection in Bangladesh was reported on March 8, 2020 [4]. Bangladesh was facing an increasing risk of imported and local COVID-19 hotspots. As of November 18, 2021, Bangladesh has reported 1,573,214 positive cases and 27,934 deaths from 10,653,924 lab tests [5]. Quick and accurate detection of virus-infected individuals is important to minimize the spread of SARS-CoV-2. The most sensitive and specific assay for detecting SARS-CoV-2 is the real-time quantitative reverse transcription-polymerase chain reaction (RT-qPCR) [6,7]. Although numerous COVID-19 RT-qPCR kits are widely available currently, an impartial evaluation of these products has not been conducted yet to facilitate the implementation of accurate testing in a diagnostic sector crowded with new tests. To battle the COVID-19 pandemic, accurate detection tests for SARS-CoV-2 are crucial. During this emergency, several companies developed diagnostic kits for detecting SARS-CoV-2 within a short time by compromising on kit sensitivity and specificity [8].

This study aimed to compare and assess the detection performance of three SARS-CoV-2 nucleic acid detection kits for the simultaneous detection of samples collected from COVID-19 patients. In-house clinical verification of innovative RT-PCR kits is needed following their introduction. This research will aid in determining the efficacy of assorted PCR kits already in use in Bangladesh, determining the most appropriate target gene based on mutation frequency, developing a multiplex real-time RT-qPCR assay for SARS-COV-2 detection, and investigating demographic characteristics and laboratory findings of COVID-19-positive patients.$\cdot$

## Materials and methods

Collection of clinical specimens

Between August and September 2021, 150 samples (nasopharyngeal and oropharyngeal swabs in a viral transport medium [VTM]) were obtained from patients with suspected COVID-19 infection at Shaheed Suhrawardy Medical College Hospital in Dhaka, Bangladesh. The following commercially available PCR kits were used to detect SARS-CoV-2 and evaluate their sensitivity and specificity: Sansure (China), Sentinel Diagnostics (STAT-NAT^Ⓑ^, Italy), and Roche Biochem (Switzerland).

Viral RNA extraction and master mix preparation

The samples in VTM were opened in a biosafety cabinet class‐II and vortexed. Subsequently, the samples were allowed to settle for 10 minutes.

For the Sansure PCR kit, 10 μL of the specimen was pipetted into a 1.5 mL Eppendorf tube, and 10 μL sample release reagent was added into each tube. The mixture of the lysis buffer and the sample was vigorously vortexed in a 1.5 mL Eppendorf tube for 10 seconds and allowed to incubate for 10 minutes at room temperature inside a biosafety cabinet class‐II to ensure rapid virus deactivation and RNase denaturation. Then, 30 μL of PCR master mix (26 µL PCR mix + 4 µL of PCR enzyme mix) for each sample were prepared. For each sample, 20 µL of lysates were transferred into a PCR tube well. After covering each well and centrifuging at 2,000 rpm for 10 seconds, PCR tubes were placed into the PCR machine (Quant StudioTM 5, Thermo Fisher Scientific, USA) for real-time RT-qPCR amplification. Internal controls targeting the RNase *P* gene were used for supervising sample collection, sample processing, and qRT-PCR to avoid false-negative findings.

In the case of the STAT-NAT^Ⓑ^ PCR kit, the RNA extraction procedure was the magnetic bead-based automatic nucleic acid extraction system NC-15 Plus (ALPHAGENE Co. Ltd., Sengnam, Korea). Each STAT-NATⒷ Pathomag extraction kit well contained 4 μL of poly(A) RNA, 15 μL of proteinase K, 250 μL of extraction buffer I, 250 μL of extraction buffer II, and 50 μL of elution buffer. First, 200 μL of the sample was added to each extraction kit well, and wells were placed in the NC-15 Plus before starting the run. RNA extraction was completed after 23 minutes. Because the STAT-NAT^Ⓑ^ PCR kit was lyophilized, we added a buffer. To conduct real-time RT-qPCR amplification, 10 µL of extracted RNA was combined with 15 µL of the master mix. Each reaction had an endogenous internal control (IC) (human RNase P), which provided information on the system’s characteristics and ensured the lack of inhibitors of polymerase activity.

For the Roche PCR kit, RNA was extracted using an automated magnetic bead-based MagNA Pure 96 System (Roche Co. Ltd., Switzerland). Each MagNA Pure LC Total Nucleic Acid Isolation Kit well contained 6 μL of poly(A) RNA, 10 μL of proteinase K, 200 μL of extraction buffer I, 200 μL of extraction buffer II, and 40 μL of elution buffer. First, 10 μL of equine arteritis virus (EAV) control and 250 μL of the sample were added to each well. Subsequently, wells were placed in the MagNA Pure 96 System and the extraction processing was started. It took 45 minutes to complete RNA extraction. In total, 10 μL Roche master mix consisted of 4.9 µL of PCR-grade water, 4.0 µL Roche Master, 0.1 µL RT enzyme, 0.5 µL EAV control, and 0.5 µL of parameter-specific reagent (*E*/*RdRP*/*ORF1ab* genes). To perform real-time RT-qPCR amplification, 10 µL of elute RNA volume was added with the master mix. This assay uses EAV as an extraction control to ensure the quality of the RNA extraction phase and amplification.

RT-qPCR analysis

Three different PCR kits were used for the qualitative detection of SARS-CoV-2 following the manufacturer’s instructions (Table 1). To exclude the possibility of equipment performance influencing the results, all PCR reactions were conducted on the same RT-PCR machine (Quant StudioTM 5, Thermo Fisher Scientific, USA) under the following conditions:

(a) The Sansure PCR kit included an RT step at 50°C for 30 minutes, followed by a one-minute cDNA pre-denaturation step at 95°C. This was followed by 45 cycles of 15 seconds for denaturation at 95°C and 30 seconds for annealing (with fluorescence monitoring) and elongation at 60°C. Finally, 10 seconds at 25°C for the instrument to cool down.

(b) The STAT-NAT^Ⓑ^ PCR kit included an RT stage at 50°C for 10 minutes, followed by a two-minute cDNA pre-denaturation step at 95°C. This was followed by 10 cycles of 15 seconds at 95°C for denaturation and 30 seconds at 58°C for annealing (without fluorescence monitoring) and elongation. Subsequently, 35 cycles of 15 seconds at 95°C for denaturation and 30 seconds at 58°C for annealing (with fluorescence monitoring) and elongation.

(c) The Roche PCR kit included an RT step at 55°C for 5 minutes, followed by a cDNA pre-denaturation step at 95°C for five minutes. This was followed by 44 cycles of five seconds at 95°C for denaturation and 15 seconds at 60°C for annealing (with fluorescence monitoring) and elongation. Finally, the instrument was allowed to cool down for 30 seconds at 40°C.

Table 1 presents a summary of real-time RT-qPCR kits used in this study and their rules of interpretation.

**Table 1:** Summary of real-time RT-qPCR kits used and their rules of interpretation. LOD: limit of detection; PCR: polymerase chain reaction; Ct: cycle threshold; RT-qPCR: quantitative reverse transcription-polymerase chain reaction

Assay	Target genes	Internal control	RNA (template) volume (μL)	Analytical sensitivity (LOD)	Running time of PCR	Rules for interpretation of results			
Sansure, China	*ORF1ab*, *N*	RNase P	10	200 copies per mL	97 minutes	*ORF1ab* sigmoidal amplification curve and Ct ≤ 40	*N* gene sigmoidal amplification curve and Ct ≤ 40	Status of result	
						+	+	Positive	
						+	−	Positive	
						−	+	Positive	
						−	−	Negative	
Roche, Switzerland	*RdRp*, *E*	RNase P	10	10 genomic copy equivalents	70 minutes	*RdRp* sigmoidal amplification curve and Ct ≤ 40	*E* gene sigmoidal amplification curve and Ct ≤ 35	Status of result	
						+	+	Positive	
						-	+	Negative	
STAT-NAT^Ⓑ^, Italy	*ORF1ab*, *RdRp*, *E*	RNase P	10	500 copies per mL	80 minutes	*ORF1ab* sigmoidal amplification curve and Ct ≤ 35	*RdRp* sigmoidal amplification curve and Ct ≤ 35	*E* gene sigmoidal amplification curve and Ct ≤ 30	Status of result
						+	+	+	Positive
						If only one target is positive		+	Positive
						−	−	+	Negative

Analysis of the results

For all three kits, the test findings were interpreted according to the manufacturer’s instructions. A positive result for Sansure was a sigmoidal amplification curve in *ORF1ab* and/or *N* gene with Ct of ≤40. For this test, the RT-qPCR result was considered positive if: (1) only the *ORF1ab* gene was positive, (2) only the *N* gene was positive, or (3) both *ORF1ab* and *N* genes were positive at the same time.

A specimen was positive for the STAT-NAT^Ⓑ^ kit if the sigmoidal amplification curve in the *E* gene had a Ct of ≤30 and the sigmoidal amplification curve in the *ORF* and *RdRp* gene had a Ct of ≤35. For this test, the RT-qPCR result was considered positive if: (1) both *ORF* and *E* genes were positive at the same time, (2) both *RdRp* and *E* genes were positive at the same time, or (3) *ORF*, *RdRp*, and *E* genes were positive at the same time.

A specimen was positive for the Roche PCR kit if the sigmoidal amplification curve in the *E* gene had a Ct of ≤35 and the sigmoidal amplification curve in the *ORF* and *RdRp* genes had a Ct of ≤40. The result was considered positive if both *RdRp* and *E* genes were positive at the same time.

Statistical analysis

As the data were normally distributed, the arithmetic mean with standard deviation was used. Categorical variables are presented as numbers and percentages. To compare numerical variables with normal distributions between three independent samples, a one-way analysis of variance with Tukey post hoc test was used. Fisher’s exact test was used to compare the frequencies of categorical variables within independent samples. Additionally, diagnostic accuracy was calculated by comparing all three methods. P-values of <0.05 were considered significant. The analysis was conducted using SPSS version 21.0 (IBM Corp., Armonk, NY, USA). We analyzed the receiver operating characteristic (ROC) curve to measure the area under the curve (AUC).

## Results

In this cross-sectional study, a total of 150 randomly selected COVID-19 patients were enrolled. The mean age of the participants was 49.64 ± 18.30 years. Overall, 51.33% (77) were males, and 48.67% (73) were females. Presenting symptoms of the patients are listed in Table 2.

**Table 2:** Presenting symptoms of the study participants.

Symptoms	Positive n = 72 (%)	Negative n = 78 (%)
Headache	3 (4.17)	12 (15.38)
Cough	33 (45.83)	35 (44.87)
Fever	18 (25.00)	26 (33.33)
Breathlessness	35 (48.61)	16 (20.51)
Not applicable	7 (9.72)	9 (11.54)

The following three PCR (RT-qPCR) kits were used: Sansure, STAT-NAT^Ⓑ^, and Roche Biochem. Table 3 summarizes the results obtained using all three kits.

**Table 3:** Comparison of the three RT-qPCR kits used for testing the 150 specimens. RT-qPCR: quantitative reverse transcription-polymerase chain reaction

RT-qPCR kit	Positive, n (%)	Negative, n (%)
Sansure	24 (16.0%)	126 (84.0%)
STAT-NAT^Ⓑ^	61 (40.7%)	89 (59.3%)
Roche	72 (48.0%)	78 (52.0%)

Table 4 presents the Ct values for all real-time RT-qPCR kits and compares them. When a gene was considered positive, the average Ct value was determined using the arithmetic mean of all Ct values (single or multiple-gene RT-qPCR positivity). The average Ct values for *ORF1ab* gene amplification obtained using the STAT-NAT^Ⓑ^ RT-qPCR kit were significantly lower (p < 0.001) than those obtained using the Sansure method. The average Ct values obtained using the STAT-NAT^Ⓑ^ RT-qPCR kit were considerably lower than the Roche Biochem kit (p < 0.001). The STAT-NAT^Ⓑ ^Ct values for *RdRp* gene amplification were significantly lower than those obtained using the Roche Biochem kit (p < 0.001).

**Table 4:** Comparison of Ct values obtained using the Sansure, STAT-NATⒷ, and Roche RT-qPCR kits. *Significance level of 0.05 according to Tukey HSD post-hoc test. RT-qPCR: quantitative reverse transcription-polymerase chain reaction; Ct: cycle threshold; SD: standard deviation; HSD: honestly significant difference

Gene	Ct value (mean ± SD)			P-value*		
	Sansure	STAT-NAT^Ⓑ^	Roche	Sansure vs. STAT-NAT^Ⓑ^	Sansure vs. Roche	STAT-NAT^Ⓑ^ vs. Roche
*N* gene	32.85 ± 3.65	/	/	NA	NA	NA
*ORF1ab*	36.67 ± 2.75	22.78 ± 5.90	/	<0.001	NA	NA
*E* gene	/	20.74 ± 5.82	26.92 ± 6.57	NA	NA	<0.001
*RdRp*	/	23.87 ± 6.23	30.03 ± 6.77	NA	NA	<0.001

The association between Ct values obtained using two different RT-qPCR methods is shown in Table 5. There was a strong positive significant correlation between the Ct values obtained using the STAT-NAT^Ⓑ^ and Roche Biochem RT-qPCR kits. This finding indicated a high degree of reliability between methods.

**Table 5:** Correlation matrix for the Ct values obtained using STAT-NATⒷ and Roche RT-qPCR kits. RT-qPCR: quantitative reverse transcription-polymerase chain reaction; Ct: cycle threshold

RT-qPCR kit	*E* gene		*RdRp*	
	STAT-NAT^Ⓑ^	Roche	STAT-NAT^Ⓑ^	Roche
STAT-NAT^Ⓑ^	1	r = 0.956, p = <0.0001	1	r = 0.927, p = <0.0001
Roche		1		1

**Figure 1:**
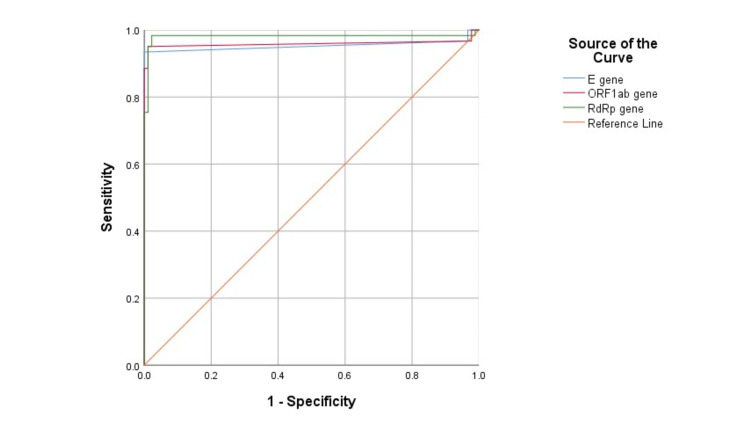
ROC curve of Ct values for E, ORF1ab, and RdRp genes of the STAT-NATⒷ PCR kit. ROC: receiver operating characteristic; Ct: cycle threshold; PCR: polymerase chain reaction

Table 6 summarizes the test performance of all three kits. In general, the STAT-NAT^Ⓑ^ and Roche RT-qPCR kits demonstrated high diagnostic sensitivity for detecting SARS-CoV-2-positive patients. In summary, Roche and STAT-NAT^Ⓑ^ had higher sensitivity than Sansure, while STAT-NAT^Ⓑ^ and Sansure had the highest specificity. The Roche and STAT-NAT^Ⓑ^ kits had the highest sensitivity and were the most accurate. The likelihood ratio for a positive test result (LR+) was more significant for the STAT-NAT^Ⓑ^ than for the Sansure or Roche kits. Table 7 presents the AUC, standard error, P-value and asymptotic 95% CIs of Ct values for *E*, *ORF1ab*, and *RdRp* genes of the STAT-NAT^Ⓑ^ PCR kit. Figure 1 shows the ROC curve of Ct values for E, ORF1ab, and RdRp genes of the STAT-NATⒷ PCR kit.

**Table 6:** Determination of test accuracy using Sansure, STAT-NATⒷ, and Roche RT-qPCR kits. CI: confidence interval; Sn: sensitivity; Sp: specificity; LR+: likelihood ratio for a positive test; LR-: likelihood ratio for a negative test; RT-qPCR: quantitative reverse transcription-polymerase chain reaction

	Diagnostic accuracy with 95% CI				
RT-qPCR kit	Sn (%)	Sp (%)	Overall acuracy (%)	LR+	LR-
Sansure	62.24 (51.88-71.84)	96.74 (90.77-99.32)	78.95 (72.46-84.51)	19.09 (6.21-58.72)	0.39 (0.30-0.50)
STAT-NAT^Ⓑ^	83.56 (73.05-91.21)	97.80 (92.29-99.73)	91.46 (86.09-95.25)	38.02 (9.62-150.28)	0.17 (0.10-0.28)
Roche	96.83 (89.00-99.61)	89.00 (81.17-94.38)	93.17 (88.10-96.54)	8.80 (5.03-15.40)	0.04 (0.01-0.14)

**Table 7:** AUC, standard error, P-value, and asymptotic 95% CIs of Ct values for E genes, ORF1ab genes, and RdRp genes of the STAT-NATⒷ PCR kit. AUC: area under the curve; CI: confidence interval; Ct: cycle threshold; PCR: polymerase chain reaction

Gene	Cut-off value	AUC	Standard error	P-value.	Asymptotic 95% CI	
					Lower bound	Upper bound
E	30	0.952	0.025	<0.001	0.903	1.000
ORF1ab	32	0.959	0.024	<0.001	0.912	1.000
RdRp	32	0.981	0.016	<0.001	0.949	1.000

**Figure 2:**
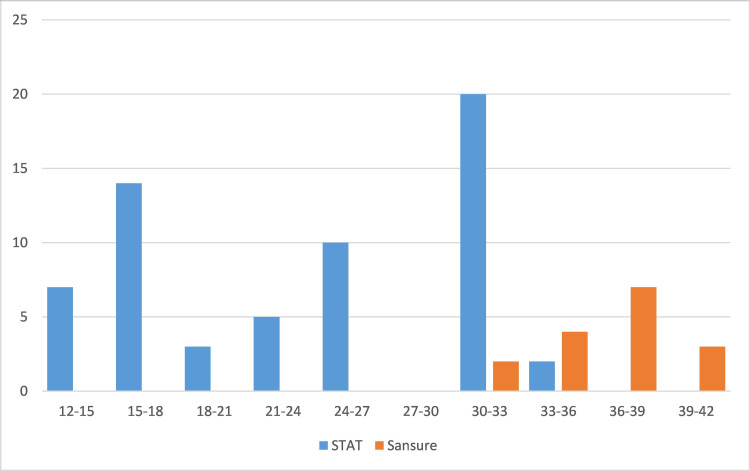
Ordination of Ct values corresponding to the amplification of the ORF1ab gene using STAT-NATⒷ and Sansure RT-qPCR kits. RT-qPCR: quantitative reverse transcription-polymerase chain reaction; Ct: cycle threshold

**Figure 3:**
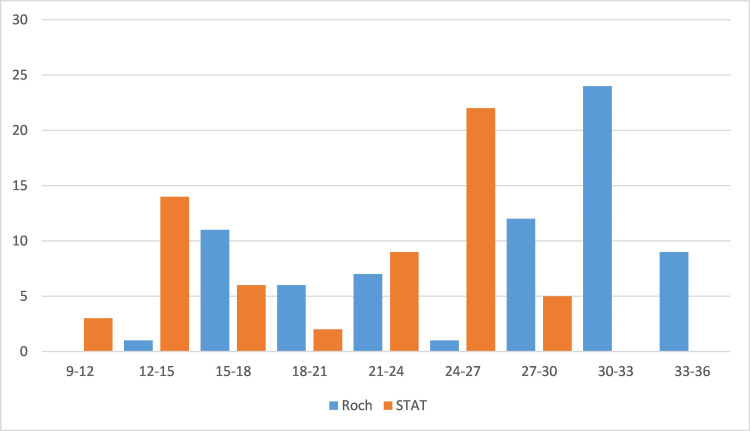
Ordination of Ct values corresponding to the amplification of the E gene using Roche and STAT-NATⒷ RT-qPCR kits. RT-qPCR: quantitative reverse transcription-polymerase chain reaction; Ct: cycle threshold

**Figure 4:**
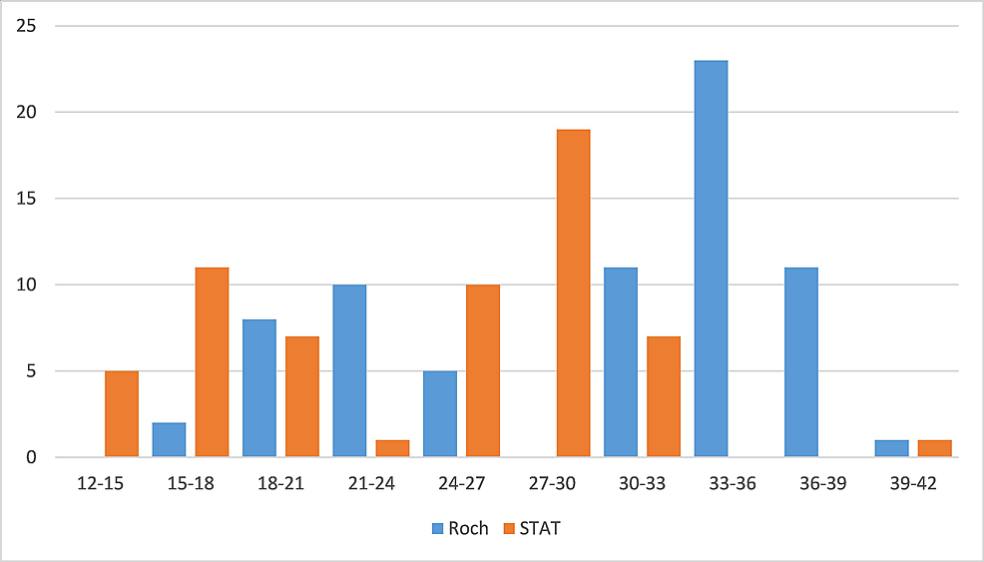
Ordination of Ct values corresponding to the amplification of the RdRp gene using Roche and STAT-NATⒷ RT-qPCR kits. RT-qPCR: quantitative reverse transcription-polymerase chain reaction; Ct: cycle threshold

Figures 2-4 illustrate the ordination of Ct values for the *ORF1ab* and *N* genes using the Sansure Biotech, STAT-NAT^Ⓑ^, and Roche kits.

## Discussion

To control the COVID-19 pandemic and ensure proper management of patients, rapid and accurate detection of SARS-CoV-2 is crucial [9]. However, using insensitive and nonspecific SARS-CoV-2 PCR test kits can lead to false-negative and false-positive test results, respectively [10,11].

To evaluate the sensitivity and specificity of three commercially available real-time RT-qPCR kits, namely, Sansure, STAT-NAT^Ⓑ^, and Roche, between August and September 2021, we collected 150 upper and lower respiratory tract samples from participants aged 49.64 ± 18.30 years. Symptoms such as headache, fever, and breathlessness significantly differed for positive and negative samples; however, cough was a common complaint. Compared to the Sansure RT-PCR kit, there were 37 more positive samples using the Roche RT-PCR kit and 48 more positive samples using the STAT-NAT^Ⓑ^ kit. The STAT-NAT^Ⓑ^ RT-qPCR kit could detect the* ORF1ab* gene sensitively (p < 0.001) than the Sansure kit. STAT-NAT^Ⓑ^ could also detect *E* gene and *RdRp* gene more sensitively (p < 0.001) than Roche. Our findings show that the STAT-NAT^Ⓑ^ kit is more sensitive than the other two, as indicated by their Ct values of respective genes. Regarding specificity, STAT-NAT^Ⓑ^ (95% Cl: 92.29-99.73%) showed more accuracy than Sansure (95% Cl: 90.77-99.32%) and Roche (95% Cl: 81.17-94.38%). The AUC for *E*, *ORF1ab*, and RdRp genes of the STAT-NAT^Ⓑ^ PCR kit was 0.952, 0.959, and 0.981, respectively.

We observed a Ct value of >28 for Roche and >20 for STAT-NAT^Ⓑ^. The detection rate of the *ORF1ab* gene was significantly higher for STAT-NAT^Ⓑ^ than the Sansure PCR kit (Figure 2). However, the detection rate of the *E* gene and *RdRp *gene was not significantly different in the STAT-NAT^Ⓑ^ and Roche PCR kits (Figures 3, 4). For Sansure, the positive rate increased due to the detection of the *N* gene, but the detection rate of the *ORF1ab *gene was low. As a larger amplitude of mRNAs for the *N* gene increases the sensitivity of the SARS-CoV-2 detection kit [12,13], the inclusion of the *N* gene as a target increases the positivity rate detected by the Sansure PCR kit.

In Bangladesh, several variants of SARS-CoV-2 have been identified. The alpha variant was first identified on January 6, 2021, at the same time as the Wuhan-like variant was identified in Bangladesh. The alpha variant was identical to UK SARS-CoV-2 Variant B.1.1.7 [14]. The beta variant of SARS-CoV-2 was then detected on March 16, 2021, in Bangladesh. Following the beta variant, the delta variant, the most detrimental variant originating from India, was identified on May 8, 2021 [15]. The lambda variant, originating from South America, was first identified in Bangladesh in August 2021 [16]. The delta variant of SARS-CoV-2 was then detected in June 2021 in Bangladesh [17]. Being a middle-income country, the government of Bangladesh could not afford frequent variant identification. Therefore, we can assume that these variants entered Bangladesh long before their identification. These factors delayed the development of the SARS-CoV-2 detection PCR kit. Even after identifying different SARS-CoV-2 variants at different times, some companies did not develop PCR kits for detecting new variants. Consequently, the sensitivity and specificity of COVID-19 diagnostic tests were decreased due to the lowering of the test positivity rate.

Banko et al. performed a comparative study with three PCR kits, namely, Sansure Biotech (Sansure Biotech Inc., China), GeneFinderTM (OSANG Healthcare Co., Seongnam, Korea), and TaqPathTM (Thermo Fisher Scientific, Waltham, MA, USA). They showed that the Sansure Biotech assay had greater diagnostic performance than the other two PCR kits [8]. Moore et al. performed another comparative study with two PCR kits, namely, Sansure Biotech and BioGerm (Shanghai, China). They showed that the BioGerm PCR kit had higher diagnostic performance than that of the Sansure PCR kit [9]. In our study, we found that STAT-NAT^Ⓑ^ is a better diagnostic RT-qPCR kit compared to Sansure and Roche for detecting SARS-CoV-2.

Overall, approximately 150 molecular laboratories in Bangladesh to diagnose COVID-19 operated by both governmental and non-governmental organizations have been established within a year [5]. Establishing and maintaining these laboratories within a short period was very challenging. A collaboration of non-profit national and international organizations with the government allowed in overcoming obstructions by arranging massive training programs. However, the threat of cross-contamination remains in real-time RT-qPCR molecular laboratories due to a lack of expert personnel and advanced facilities [18].

There are some limitations of our study. First, the lack of gold standards results in invalid analytical sensitivity. Second, RNA extraction procedures were not identical for all three real-time RT-qPCR kits. While an automated magnetic bead-based method was used for RNA isolation for STAT-NAT^Ⓑ^ and Roche RT-qPCR kits, Sansure used a lysis extraction procedure. Third, all target genes were not identical among the three kits.

## Conclusions

We evaluated the detection performance regarding the sensitivity and specificity of three commercially available real-time RT-qPCR kits to detect SARS-CoV-2. The sensitivity of Roche and STAT-NAT^Ⓑ^ PCR kits for the identification of SARS-CoV-2 was higher than that for the Sansure PCR kit. However, the specificity of STAT-NAT^Ⓑ^ and Sansure was higher than the Roche PCR kit. In summary, STAT-NATⒷ showed better diagnostic performance than Sansure and Roche in detecting SARS-CoV-2.
